# First characterization of toxic alkaloids and volatile organic compounds (VOCs) in the cryptic dendrobatid *Silverstoneia punctiventris*

**DOI:** 10.1186/s12983-021-00420-1

**Published:** 2021-08-26

**Authors:** Mabel Gonzalez, Pablo Palacios-Rodriguez, Jack Hernandez-Restrepo, Marco González-Santoro, Adolfo Amézquita, Andrés E. Brunetti, Chiara Carazzone

**Affiliations:** 1 Department of Chemistry, Universidad de los Andes, Bogotá, AA 4976 Colombia; 2 Department of Biological Sciences, Universidad de los Andes, Bogotá, AA 4976 Colombia; 3 Laboratorio de Genética Evolutiva, Instituto de Biología Subtropical (CONICET - UNaM), Facultad de Ciencias Exactas, Universidad Nacional de Misiones, N3300 Posadas, Argentina; 4 Departamento de Ciências Biomoleculares, Faculdade de Ciências Farmacêuticas de Ribeirão Preto, Universidade de São Paulo, 14040-903 Ribeirão Preto, SP Brazil

**Keywords:** HS-SPME/GC-MS, Odour, Chemical communication, Anti-predatory signal, Unpalatability, Repellent, Poison frog

## Abstract

**Background:**

Poison frogs are known for the outstanding diversity of alkaloid-based chemical defences with promising therapeutic applications. However, current knowledge about chemical defences in Dendrobatoidea superfamily has two sources of bias. First, cryptic, brown-colored species have been neglected in comparison to those conspicuously colored, and second, there has been little interest in characterizing metabolites other than alkaloids mediating defensive functions. In an effort to contribute to fill the gap of knowledge about cryptic species and broadening the spectrum of compounds analyzed we have applied head-space solid phase microextraction coupled to gas chromatography and mass spectrometry (HS-SPME/GC-MS) for extracting amphibian alkaloids and volatile organic compounds (VOCs) from *Silverstoneia punctiventris*.

**Results:**

Using the skin from 8 specimens in 4 biological replicates we have found 33 different compounds. Twenty of them were classified as VOCs into 15 chemical classes including alkanes, alcohols, carbonyl compounds, methylpyridines, benzothiazoles, N-alkylpyrrolidines, pyrazines, and sesquiterpenoids, some of which were previously reported as repellents, defence compounds or defence pheromones in other organisms, and as sex pheromones in a treefrog. Interestingly, six of the remaining compounds were identified as alkaloids previously reported in other toxic/unpalatable dendrobatid frogs.

**Conclusions:**

This is the first report of alkaloids and VOCs found in the *Silverstoneia* genus, which has been assumed for decades as non-chemically defended. This study establishes HS-SPME/GC-MS as a new application for a simultaneous approach to amphibian alkaloids and VOCs in poison frogs while opens up new research questions to assess the co-occurrence of both type of compounds and to investigate the evolutionary significance of a defence gradient that includes olfactory avoidance, unpalatability, and toxicity in dendrobatids. In addition, our results show that amphibian alkaloids could have a dual function (olfactory at distance, taste by contact) never explored before neither in *Silverstonaeia* nor in any other dendrobatid species.

**Supplementary Information:**

The online version contains supplementary material available at 10.1186/s12983-021-00420-1.

## Background

Alkaloids are basic nitrogen-containing compounds mostly described in plants to arguably deter herbivores, and can be sequestered by many invertebrate and vertebrate animals to reduce the probability of being attacked [1–4]. They have been documented in a wide array of phylogenetically distant anuran families: Bufonidae (*Melanophryniscus*) [5], Eleutherodactylidae (*Eleutherodactylus*) [6], Mantellidae (*Mantella*) [7], Myobatrachidae (*Pseudophryne*) [8], and Dendrobatidae (Dendrobatoidea sensu Grant et al. 2017) [9]. Over 900 alkaloids have been characterized in amphibians [10–12], highlighting the chemical diversity found in Neotropical dendrobatid poison frogs which include more than 500 compounds classified in the following lipophilic families: batrachotoxins, histrionicotoxins, gephyrotoxins, pumiliotoxins, allopumiliotoxins, homopumiliotoxins, decahydroquinolines, pyrrolizidines, indolizidines, quinolizidines, lehmizidines, pyrrolidines, piperidines, tricyclics and pyridinic alkaloids [10]. Additionally, hydrophilic alkaloids, such as tetrodotoxins, have also been found in two dendrobatid species [13, 14]. Current evidence suggests that most alkaloids are sequestered from dietary sources [15] and in some cases even metabolically transformed [16]. Some pumiliotoxins for example are derived from mites [17] or ants [18], whereas spiropyrrolizidines are sequestered from mites and millipedes [19, 20]. Many of these alkaloids possess toxic [2] or unpalatable function [21], but the evolutionary significance of toxicity versus unpalatability needs to be further explored [22].

From the organism perspective, most of the alkaloids discovered in Dendrobatoidea belong to the conspicuously-colored genera in the lineage Dendrobatidae. A second lineage, called Aromobatidae, is mostly composed of cryptic and presumably palatable frogs [23, 24]. Cryptic species comprise approximately two thirds of the species of this superfamily and belong to the genera *Allobates*, *Anomaloglossus*, *Rheobates*, *Aromobates* and *Mannophryne* (from Aromobatidae), and *Colostethus*, *Silverstoneia*, *Epipedobates*, and *Hyloxalus* (from Dendrobatidae) [22]. Only some species from the genera *Aromobates, Colostethus*, *Epipedobates*, and *Hyloxalus* are recognized to be chemically defended [22]. However, looking carefully it becomes evident there is a gap of information about the alkaloid profile of most dendrobatids, because only 12% (24 out of approximately 200 cryptic species) have been chemically surveyed and most of them from very few specimens. The absence of alkaloids of the remaining majority of species has been extrapolated based on these scarce analyses.

From the chemical perspective, chemical defences other than alkaloids have been largely overlooked in dendrobatids. Whether non-alkaloid metabolites detected in dendrobatids have an anti-predatory function is an unresolved question. Some of the compounds that deserve further attention include biogenic amines, bufadienolides, a dipeptide called carnosine (detected in *Phyllobates*) [2], deltorphins, bufogenins, bufotenins, (putatively identified in *P. vitattus*) [25], and presumably defensive malodorous compounds perceived in *Aromobates nocturnus* (without successful chemical characterization) [26]. In particular, different volatile organic compounds (VOCs) have been characterized in other amphibians such as pelodryadids [27], mantellids [28], hyperoliids [29], and hylids [30, 31]. Because some of these compounds have been associated to defensive functions [27, 30] a comprehensive chemical characterization that includes diverse compound types such as alkaloids and VOCs can improve our understanding of the evolution of chemical defences within the superfamily. Additionally, ﻿alkaloids and other non-alkaloid compounds found in dendrobatids could stimulate olfactory or gustatory channels, or both, as it has been shown with other chemical defences from invertebrates [32].

Cryptic species usually rely on their visual camouflage for defence against predators [33], but some examples of cryptically colored dendrobatids demonstrate the presence of chemical compounds as an additional defending strategy. For instance, the potent tetrodotoxin (TTX) has been found in *Colostethus panamensis* [13] and *C. ucumari* [34], two species with mainly brown coloration. *Aromobates nocturnus* is known to release an intriguing pungent (mercaptan) odor that could work as a defensive mechanism at a distance (non-contact) [26]. Using mice bioassays, it was shown that *Allobates femoralis*, another cryptic species, contains unidentified natural products (not necessarily alkaloids) that affect mice well-being as inferred from their behavior [35, 36], but see [37]. In addition to the lack of consensus over whether some species are toxic or not, it is worth noting that most chemical characterization in dendrobatid species was performed several years ago using pools of dozens of individuals because of the low sensibility of analytical methods [2, 10, 15, 38–45]. Thus, current technological advances in analytical methods may provide new insights about chemical defence mechanism in cryptic species, including marked odorous compounds that have been chemically elusive [26].

Aiming to characterize VOCs within the background of defence mechanism against predators in dendrobatids and to expand the knowledge on chemical compounds found in cryptic species, we have selected *Silverstoneia punctiventris* and HS-SPME/GC-MS to explore their chemical profile*.* Unlike most species from the Dendrobatidae lineage, this species has been historically presumed to be cryptic. Only two out of the eight recognized species in *Silverstoneia* have been chemically characterized, but failure in finding alkaloids led the conclusion that the complete genus is not chemically defended [22, 46–48]. Notably, many of these studies referenced J. W. Daly pers. com., but only results from a single 11th year old skin have been published [46]. In addition, *S. punctiventris* was selected because we perceived some particular smells after handling some specimens. We chose HS-SPME/GC-MS because it allows simultaneous recovery of alkaloids and VOCs. In this document we will describe separately amphibian alkaloids and VOCs, taking into account that amphibian alkaloids are already known to be toxic or unpalatable, whereas the possible anti-predator effect of some VOCs is still unknown.

## Results

Using HS-SPME/GC-MS in eight specimens of *S. punctiventris* we found 33 different compounds, seven of which were not annotated with available information in chemical libraries (Table 1). Comparison between manual and automatic annotation from The Global Natural Products Social Networking (GNPS) was performed for all 26 annotated compounds finding that 25 of them have a coincident annotation (Additional file 2). One alkane, four alcohols, three carbonyl compounds, and one methylpyridine, were the first to be eluted. One benzothiazole, one N-alkylpyrrolidine, one pyrazine, five indolizidines, one quinolizidine, one coumarine derivative, and three sesquiterpenoids were observed at higher retention times (Table 1). There was a great variability between samples in the presence and intensity of each compound, which was particularly evident in compounds detected at low intensities. Twenty-five compounds were detected in replicate 1 (*S. punctiventris* 1 + 2), 19 in replicates 2 and 3 (*S. punctiventris* 3 + 4 and *S. punctiventris* 5 + 6), and 22 in the replicate 4 (*S. punctiventris* 7 + 8).

**Table 1:** Volatile profiles of *Silverstoneia punctiventris* using HS-SPME/GC-MS. IUPAC nomenclature or amphibian alkaloid name are presented, in addition to chemical class/subclass following Classyfire taxonomy or alkaloid family according to Daly et al. database [10], respectively. The retention time in minutes and peak area of each compound has also been specified for each replicate

Compound	Class/subclass Alkaloid family	Rt (min)	Replicate				BF
			***1***	***2***	***3***	***4***	
3-methylpentane	Alkanes	2.27	705,879		1,124,419		nd
3-methylbutan-1-ol	Alcohols and polyols	4.47	372,701	314,028	1,120,769	924,992	D ^2^
hexanal	Carbonyl compounds	6.05	214,369	319,896	360,387	235,433	D ^2^
2,4,6-trimethylpyridine	Methylpyridines	11.52	1,075,816	742,879	514,956	1,386,495	nd
2-ethylhexan-1-ol	Fatty alcohols	12.57	926,002	504,742	387,674	353,196	nd
N,N-dimethyl-1-phenylmethanamine	Phenylmethylamines	12.89	1,372,753	718,613	1,524,860	6,240,599	nd
octan-1-ol	Fatty alcohols	13.72		166,013			R ^2^
nonanal	Carbonyl compounds	14.65	443,257	1,896,061	1,554,323	1,845,908	nd
1,2-dimethoxybenzene	Methoxybenzenes	15.85	616,333			545,145	D ^2^
nonan-1-ol	Fatty alcohols	16.61		193,102	117,130		nd
**167E**	3,5-I	17.43				305,129	t B* ^1^
decanal	Carbonyl compounds	17.63	278,696	700,648	1,604,149	1,134,669	D ^2^
1,2-benzothiazole	Benzothiazoles	18.25	144,183	74,430	800,979	136,283	nd
Unknown1		18.55				75,485	–
3,5-dimethyl-2-(2-methylbutyl)pyrazine	Pyrazines	18.61	4,599,780	276,403	322,721		DP ^1^
Unknown2		18.92	8,629,722				–
Unknown3		19.18	31,992,502		667,980		–
Unknown4		19.86	1,087,042				–
1,2,2-triethylpyrrolidine	N-alkylpyrrolidines	20.01			229,410		R ^1^
Unknown5		20.49		213,285			–
**207I**	1,4-Q	20.59	511,736				B* ^1^
(3-hydroxy-2,4,4-trimethylpentyl) 2-methylpropanoate	Carboxylic acid derivatives	22.79	463,512	1,601,188	1,534,685	1,913,640	nd
**277E**	5,6,8-I	23.09	659,662		515,089	149,741	B* ^1^
4-(2,6,6-trimethylcyclohexen-1-yl)butan-2-one	Sesquiterpenoids	24.77	499,394	178,155	394,571	620,594	nd
(E)-4-(2,6,6-trimethylcyclohexen-1-yl)but-3-en-2-one	Sesquiterpenoids	26.18	298,444	231,400	597,650	1,081,089	nd
**223AB** (5E,9Z)	3,5-I	27.08	3,807,696			8,685,370	t B* ^1^
**223AB** (5E,9E)	3,5-I	27.49	264,366			618,971	t B* ^1^
**223AB** (5Z,9Z)	3,5-I	27.96	650,603			1,623,468	t B* ^1^
Unknown6		28.52		536,527		556,661	–
tetradecanal	Fatty aldehydes	29.76		460,880			R ^2^
Unknown7		30.24	443,555	387,061	427,026	783,730	–
N-butyl-N-(2-oxochromen-3-yl)acetamide	Coumarins and derivatives	31.02	864,020			2,087,588	nd
3-methyl-4-(2,6,6-trimethylcyclohexen-1-yl)but-3-en-2-one	Sesquiterpenoids	31.34	403,695	212,268	398,450	789,925	nd

*Abbreviations*: *3,5-I* 3,5-Disubstituted indolizidines, *1,4-Q* 1,4-disubstituted quinolizidines, *5,6,8-I* 5,6,8-Trisubstituted indolizidines, *BF* Behavioral function, *nd* not determined, *t* low toxicity, B* Presumed bitter, *D* Defence substance, *DP* Defence pheromone, *R* Repellent, ^1^ Defensive/antipredator properties measured in the class/subclass/alkaloid family, not with specific chemical structure, ^2^ Behavioral test with organisms other than amphibians

### Amphibian alkaloids

**Fig. 1:**
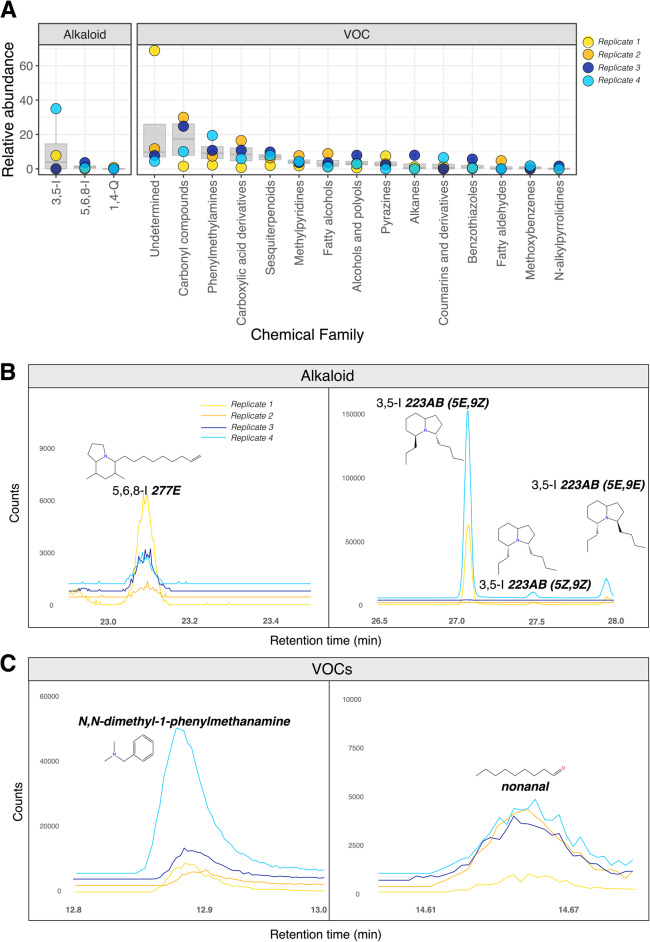
Inter-replicate variation of the compounds extracted from *Silverstoneia punctiventris.* **A. **Variation in the percentual peak areas of three amphibian alkaloid families from Daly et al. database [10] and 15 chemical VOC classes/subclasses. **B**. Extracted ion chromatograms (EIC) from fragments m/z 152 (5,6,8-I **277E**), 166 (associated with three of the isomers of 3,5-I **223AB**) corresponding to four amphibian alkaloids detected in some experimental replicates. **C**. Extracted ion chromatograms (EIC) from fragments m/z 135 (N,N-dimethyl-1-phenylmethanamine), and 98 (nonanal) corresponding to two VOCs detected on the four analyzed replicates

**Fig. 2:**
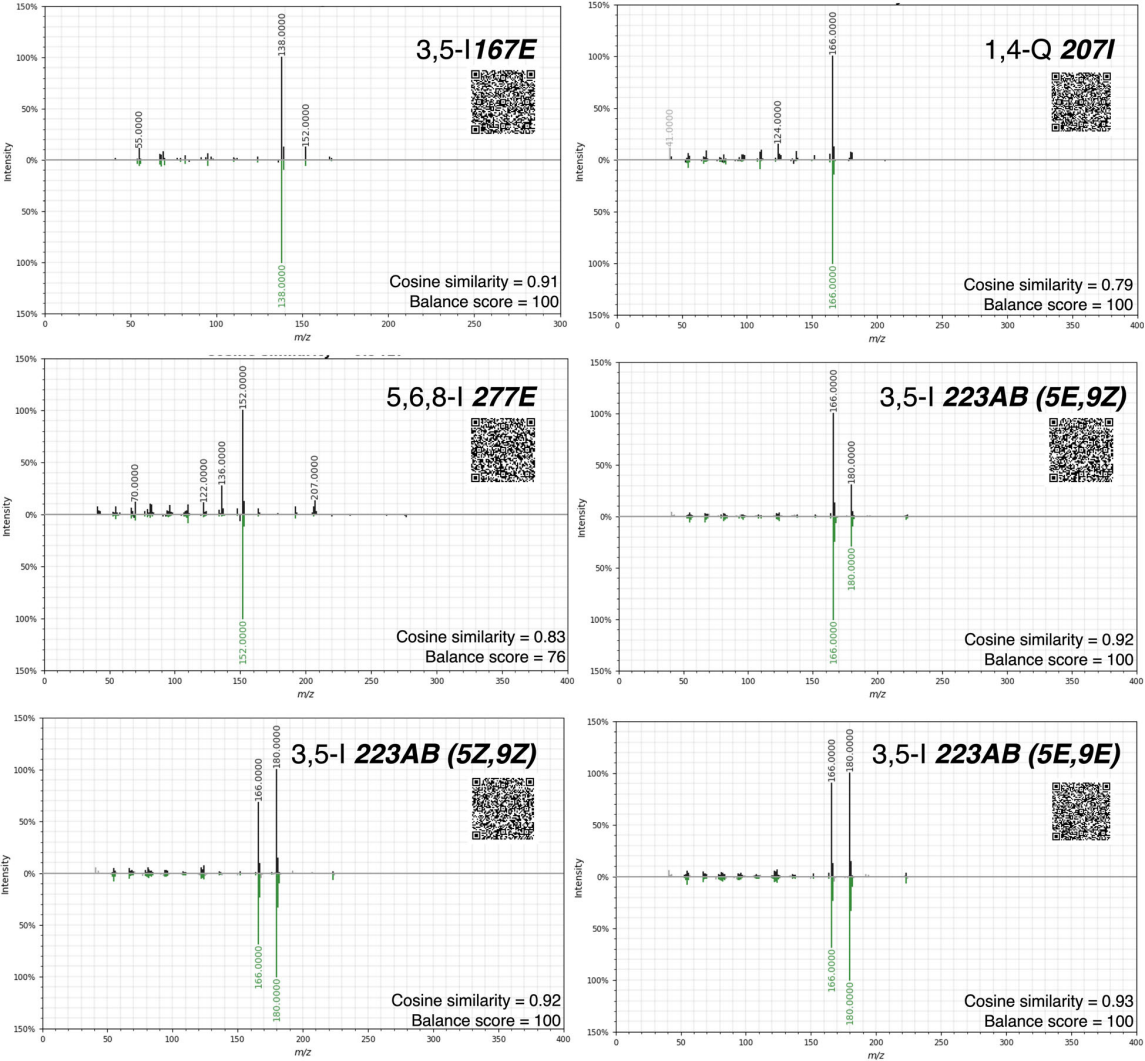
Names of putative annotated amphibian alkaloids and mirror plots comparing query experimental spectrum from *S. punctiventris* (top) and spectrum match (bottom) from GNPS libraries corresponding to each alkaloid structure. Cosine similarities and balance scores extracted from GNPS were specified for each compound. For detailed visualization of mirror plots from these alkaloids GNPS links employing the Metabolomics Spectrum Resolver Web Service [49] can be accessed through QR codes below the name of the compound

A total of six amphibian alkaloids from the Daly et al. database [10] were detected in *S. punctiventris.* We have found five indolizidine alkaloids, namely 3,5-I **167E** detected only in one replicate, three isomers of 3,5-I** 223AB** detected in two replicates and 5,6,8-I **277E** detected in three replicates. Quinolizidine 1,4-Q **207I **was detected in one of the replicates. Literature comparisons suggest that these compounds belong to amphibian alkaloid families that are toxic or presumably bitter and that have defensive/antipredator properties (Table 1). The summarized relative variation of each amphibian alkaloid class (Fig. 1A) demonstrates that 3,5-disubstituted indolizidines had a higher average variation, caused mainly by the high relative abundance of 3,5-I **223AB** (5*E*,9*Z*) in the replicate 4 (*S. punctiventris* 7 + 8) (Fig. 1A and Table 1). For better visualization of inter-replicate variation of alkaloids, some specific illustrating examples were plotted as EIC for the indolizidine 5,6,8-I **277E**, and three for the isomers of the indolizidine 3,5-I **223AB** (Fig. 1B). To facilitate verification of the annotated alkaloids, we depicted in Fig. 2 the mirror plots from alkaloids extracted from GNPS-GC-MS pipeline and two statistical outputs from it, cosine and balance score. The respective cosine similarities range from 0 to 1 (the higher the cosine, the higher is the reliability of putative annotation) and balance scores range from 0 to 100 (the higher the score, the higher is the quality of the deconvoluted mass spectra). All six alkaloids had cosine values higher than 0.75 and balance scores higher than 75 (Fig. 2 and Additional file 2).

### VOCs

We found some other non-amphibian alkaloids, such as 2,4,6-trimethylpyridine and 1,2,2-triethylpyrrolidine. The first one was detected in the four replicates, whereas the latter was detected in only one of the replicates. Other nitrogen-containing compounds include N,N-dimethyl-1-phenylmethanamine and 3,5-dimethyl-2-(2-methylbutyl) pyrazine, reported in four and three replicates, respectively. Some alcohols, carbonyl compounds, 1,2-benzothiazole, and three different sesquiterpenoids were detected in all four biological replicates. We also found other VOCs such as 3-methylpentane, 1,2-dimethoxybenzene, nonan-1-ol and the coumarine N-butyl-N-(2-oxochromen-3-yl) acetamide, detected in two replicates, while octan-1-ol and tetradecanal were detected only in one replicate.

Regarding the potential behavioral functions, we found that pyrrolidines were previously reported as repellents. Meanwhile, in other organisms, some specific compounds were described as defence substances or repellents, while other pyrazines than 3,5-dimethyl-2-(2-methylbutyl) pyrazine have functioned as defence pheromones (Table 1). Among chemical classes, carbonyl compounds, and undetermined compounds had a higher average abundance and variation (Fig. 1A). Analyzing the variation between replicates of two VOCs it can be observed, for example, that replicate 4 (*S. punctiventris* 7 + 8) has a higher relative abundance for N,N-dimethyl-1-phenylmethanamine, but not for nonanal (Fig. 1C).

## Discussion

Using HS-SPME/GC-MS, we have demonstrated the presence of alkaloids and VOCs in the *Silverstoneia* genus, specifically in *S. punctiventris* (Table 1). In addition to the remarkable diversity of alkaloids that dendrobatids contain, there is possibly an equally surprising diversity of VOCs still to be discovered, illustrated by 15 different chemical classes found in this single species (Fig. 2). The ecological relevance of these compounds and their function need to be explored in the coming years. However, our results along with some isolated reports in other dendrobatid species [26] support the hypothesis that alkaloids and some VOCs could have anti-predator functions in *S. punctiventris*. Some amphibian alkaloids are proven chemical defences [22], whereas VOCs in different organisms are known for mediating several ways of inter- and intra-specific chemical communication, as well as anti-predatory defence [30, 31]. Our findings demonstrate that in order to have a better understanding of the chemical ecology of dendrobatids, it is important to have a comprehensive chemical understanding of all purportedly non-aposematic/cryptic species, which currently is fairly incomplete in terms of species and type of metabolites surveyed.

### Amphibian alkaloids

Some of the alkaloids that we found in *S. punctiventris* (Table 1, Figs. 1B, 2) have been found in other dendrobatids and arthropods. The most abundant alkaloids*,* the three isomers of the indolizidine 3, 5-I **223AB** (Table 1, Figs. 1B and 2), have been found in toxic/unpalatable genera such as *Dendrobates, Phyllobates* [2], *Oophaga* [43], and the families Mantellidae [50] and Bufonidae [51]. There is no information regarding the specific toxicity of any isomer [52], but its action as a non-competitive blocker of the nicotinic receptor of acetylcholine (AChR) has been supported [53], and other 3–5-indolizidines have been reported as toxic [22, 54] (see behavioral functions in Table 1 and Additional file 2). These results are consistent with the potential of 3, 5-I **223AB** as a chemical defence in *S. punctiventris.* Mites are the dietary source of this compound in *Oophaga pumilio and O. sylvatica* [55, 56], but they have been also found in *Solenopsis* ants of the molesta group [57]. It is uncertain if mites are also the dietary source in *S. punctiventris*, but current evidence suggests a generalist diet in the related species *S. nubicola* that feed on mites and ants [48, 58].

It is important to clarify that even though defensive/antipredator properties have been documented in lipophilic and hydrophilic alkaloid families, most of the LD_50_ measurements on mice have been made with just a few compounds from each alkaloid family [22, 53, 54]. Individual measurements for each of the over 500 alkaloids are challenging because these alkaloids are very difficult to obtain. Frogs and their dietary prey are so far the only two natural sources, just a few commercial analytical standards are available, and the organic synthesis for those alkaloids are very difficult to achieve.

From a phylogenetic perspective, two other *Silverstoneia* have been surveyed for alkaloids, *S. nubicola* and *S. flotator* [59] but no alkaloids were detected according to J. W. Daly pers. com [22, 46–48]. The content of alkaloids/VOCs on the other five species from this genus is unknown. Interestingly, a dietary study from avian predators shows that they seem to avoid *S. flotator*, despite being one of the most abundant and prevalent frogs in their habitat [47]. The reasons for this avoidance were undetermined and could be unrelated to alkaloid content. Yet, the only published research that states absence of alkaloids in *S. flotator*, was obtained from a single specimen [46]. Ecological variation in the alkaloid profile obtained from *S. punctiventris* (Fig. 1A, B) could give insights for explaining the absence of alkaloids in some specimens and presence in others, at least in *Silverstoneia*. Indolizidine 3,5-I **167E**, for example was detected only in the replicate 4 (*S. punctiventris 7 + 8*), whereas the quinolizidine 1,4-Q **207I** was detected only in the replicate 1 (*S. punctiventris 1 + 2)* (Table 1). Previous studies on other toxic/unpalatable dendrobatids have demonstrated that not all specimens from the same species are equally defended [2, 12, 60–62], but in neither of these cases was there a specimen that lacked all alkaloids. Ecological variation of alkaloid profiles in cryptic frogs have received less attention than in conspicuously colored species, and automimicry (e.g., existence of non-defended prey in sympatry with defended conspecifics) [63] emerges as a likely hypothesis for explaining the high variation in alkaloid profiles of *S. punctiventris* and maybe other *Silverstoneia.*

Specimens of the genus *Epipedobates*, the sister taxon of *Silverstoneia,* share the presence of indolizidines, quinolizidines, and pyrrolidines in their skins [38, 64]. In contrast, pumiliotoxins, decahydroquinolines, histrionicotoxins [44, 64, 65], epibatidine (an analgesic 200 times more powerful than morphine), and the two additional pyridinic compounds (N-methylepibatidine and phantasmidine) [64, 66] from *Epipedobates* are absent in *Silverstoneia*. From an evolutionary perspective, our findings support the hypothesis that the ancestor of *Epipedobates* and *Silverstoneia* contained alkaloids. Although it seems that the diversity of alkaloids is higher in *Epipedobates,* it is still unclear until more studies are completed with *Silverstoneia*.

Our results add another “exception” to the classical aposematism (i.e. toxicity/unpalatability signaled by warning coloration) paradigm which states that chemical defences and conspicuous coloration appear to have been integrated at least four independent times [39, 67]. *Silverstoneia punctiventris* is a cryptically colored species that contains alkaloids, and *Silverstoneia* should not be assumed as a non-chemically defended genus anymore. The same pattern has been observed in the sister taxon *Epipedobates*, where *E. boulengeri*, one of the two cryptic species from the genus, also contains alkaloids. *E. machalilla*, on the other hand, is cryptic and lacks alkaloids [48, 65, 68]. The other remaining five species of *Epipedobates* have conspicuous coloration and contain alkaloids [2, 48, 65]. Other examples of cryptic species that contain alkaloids are *Colostethus panamensis* [13] and *C. ucumari* [34], which are the only two dendrobatids to contain tetrodotoxins, and *Hyloxalus erythromos*, which contains pumilitoxins and indolizidines [69]. These examples are more consistent with a change of paradigm that states that in some species aposematism could have evolved in a coupled manner, in others decoupled [70], and in others (like some polymorphic species) even inverted (i.e. more toxic phenotypes being less conspicuous) [71]. These contrasting scenarios demonstrate that the question about which came first, chemical defences or conspicuous coloration, should probably be considered with regards to understanding which different selection pressures operate on each species and how different forms of communication (visual, acoustic, and chemical) converge for its survival. The presence and the type of chemical compounds occurring in cryptic species could provide important insights about the evolution of aposematism. Future investigations studying correlations between chemical defences with other traits of the aposematic syndrome (including diet specialization, body mass, and metabolic rates) in cryptically colored and chemically defended species probably would raise new questions.

Besides toxicity, frogs also rely on the unpalatability function of alkaloids. This was first perceived by researchers after licking skin secretions when they began to study dendrobatids [21] and subsequently was further evidenced by ﻿chemically oriented arthropods that avoided frogs’ extracts by contact [72–74]. Alkaloid unpalatability then is an anti-predatory strategy for some arthropods that predate poison frogs [48, 75]. All six amphibian alkaloids detected in *S. punctiventris* (3,5-I **167E**, 5,6,8-I **277E**, 1,4-Q **207I**, and three isomers of 3,5-I **223AB**) have presumably a bitter taste [22] (see behavioral functions in Table 1 and Additional file 2). Behavioral experiments to test unpalatability in other predators of poison frogs such as birds, snakes, fishes, spiders [48] should be conducted. In spite of some of them being usually classified as mainly visually oriented predators, a more holistic perspective, where multimodal communication allows different predators to locate frogs or avoid them, should enhance our understanding about functions of chemical defences in dendrobatids. These experiments should incorporate quantitative measurements for determining if natural concentrations of amphibian alkaloids are effective toxic/unpalatable stimuli for avoiding predators.

### VOCs

Together with the amphibian alkaloids detected on *S. punctiventris*, we report 20 VOCs for the first time in the superfamily Dendrobatoidea (Table 1). Previous descriptions of odours in dendrobatids have been made in *Aromobates nocturnus* [26], which actually received its name for the mercaptan-like odour that it releases, but at that moment chemical analyses for characterizing the VOCs responsible for this particular smell were not possible. Interestingly, when alkaloids were discovered in the family Eleutherodactylidae [6], authors mentioned that ﻿the odour of some of the dissected eleutherodactylids reminded them of alkaloid-containing dendrobatid and mantellid species. However, no previous VOC profilings have been performed. The absence of previous VOC reports can be explained not by the fact that frogs lack these compounds, but by the fact that attention was mainly focused on looking for promising pharmaceutical applications of some dendrobatid alkaloids, such as epibatidine.

The ecological functions of VOCs found in *S. punctiventris* remain to be evaluated. However, through comparison with other organisms, 3-methylbutan-1-ol [76], hexanal [77], 1,2-dimethoxybenzene [78], and decanal [79] have been established as semiochemicals with defence functions in those organisms. In turn, octan-1-ol [80] and tetradecanal [81] have been described as repellents (see Table 1). Even though it is clear that VOCs’ functions vary a lot between organisms, these comparisons support the assumption that these compounds could be perceived efficiently by potential predators. In addition, ant- and other arthropod-repellents have been previously described in dendrobatids such as pyrrolidines (different from the structure found in *S. punctiventris*), piperidines, 5,8-disubstituted indolizidines, pumiliotoxins, allopumiliotoxins, histrionicotoxins, spiropyrrolizidines, batrachotoxins, pyridinic alkaloids, indolic alkaloids, ﻿N,N-diethyltoluamide, and mercaptan-odor [22, 45, 54, 82]. Yet, so far, the unpleasant taste of those alkaloids (except for the mercaptan-odor) was attributed as the main responsible of repellency. The high number of VOCs found in *S. punctiventris*, and the recovery of amphibian alkaloids employing a head-space technique opens up the possibility that besides the toxic and unpalatable taste of amphibian alkaloids, we should add now a possible olfactory avoidance function (at distance, not by contact) of amphibian alkaloids and newly discovered VOCs. These findings increase the complexity of possible mechanisms of chemical defence on several predators (chemically oriented and visually oriented) and at the same time broaden the diversity of compounds with possible anti-predatory functions.

One promising example of VOCs with possible anti-predator odour function are pyrazines. Different pyrazines have been also found in many aposematic chemically defended insects and it has been demonstrated that they are defence pheromones with that the ability to enhance an aversion response in birds even at a distance [83]. This suggests three possible scenarios for the case of *S. punctiventris*: (i) some VOCs could function also as defence pheromones for adverstising to predators about the presence of toxic/unpalatable alkaloids (e.g. olfactory aposematism) [84], (ii) toxic alkaloids themselves could be volatilized and smelled by predators (at a distance) to induce a repellent behavior, (iii) toxic alkaloids and VOCs could work synergically to deter olfactory predators. Other alternatives for avoiding predators could be using certain odours as a form of camouflage with their environment, and being cryptically odorous species [85]. Testing the repellency or camouflage olfactory potential of alkaloids and VOCs from *Silverstoneia* could give insights for understanding if chemical communication explains why avian predators seem to avoid *S. flotator*, despite being one of the most abundant and prevalent amphibians in their habitat [47].

From a phylogenetic perspective, VOC comparisons with other dendrobatids are currently not possible, because this is the first VOC survey within the superfamily. But, we are working on the characterization of VOC profiles from other dendrobatids as well (M. Gonzalez, A. Brunetti, A. Amézquita, M. González-Santoro, P. Palacios-Rodriguez, J. Hernandéz-Restrepo, A. Aksenov, P. Dorrestein, C. Carazzone, unpublished data). Other non-dendrobatid amphibians do contain some of the chemical classes of VOCs found in *S. punctiventris* (Fig. 1A) including sesquiterpenoids, alcohols, carbonyl compounds, and pyrazines. Sesquiterpenoids have been reported in *Litoria caerulea* [27], hyperoliids [29], *Boana pulchella*, *B. riojana* [30] and *B. prasina* [31]. These last three species also emitted carbonyl compounds and pyrazines. Other chemical classes, found in other amphibians but absent in *S. punctiventris*, include oxacines (found in *Mantidactylus multiplicatus*) [28], esters, macrolides (detected in hyperoliids) [29], and monoterpenes (emitted by *Boana pulchella*, *B. riojana*, and *B. prasina*) [30, 31]. The biological roles suggested for some VOCs described in amphibians include sexual pheromones in Mantellids [28], and probably also in *Boana prasina* (that show significant sexual semi-quantitative differences) [31], and repellency against mosquitoes in *L. caerulea* [86]. Ecological function for most of the compounds reported in other amphibians have not been studied yet. Besides anti-predator role, arthropod repellent and sexual pheromones, other ways of inter- and intra-specific chemical communication that should be taken into account in future research include alarm pheromones, kin recognition, and antimicrobial properties [85].

Some compounds from *S. punctiventris* have been found in plants, microorganisms, insects and even humans. Shared compounds with plants are 3-methylpentane, 1,2-dimethoxybenzene, nonan-1-ol, decanal, the ionones products of carotenoid degradation: dihydro-*β*-ionone (4-(2,6,6-trimethylcyclohexen-1-yl)butan-2-one) and *β*-ionone ((E)-4-(2,6,6-trimethylcyclohexen-1-yl) but-3-en-2-one), tetradecanal [87], and 1,2-benzothiazole, that have been found in mango [88]. Many microbial volatile organic compounds (mVOC) were also detected, such as 3-methylbutan-1-ol, hexanal, 2-ethylhexan-1-ol, octan-1-ol, nonanal, decanal [89], and pyrazines that at least in the frog *B. prasina* are linked to a bacterial origin [31]. Future microbiological analysis looking for ﻿*Pseudomonas sp.* strains should help to determine if 3,5-dimethyl-2-(2-methylbutyl) pyrazine from *S. punctiventris* also have a bacterial origin. 3-methylpentane [90] and 3-hydroxy-2,4,4-trimethylpentyl 2- methylpropanoate [91] have been found in human breath. In addition to microbial origin, a dietary origin of some compounds is also possible, as it occurs in other amphibians [27, 30]. The large inter-replicate variation found in the VOC profiles from *S. punctiventris* could be linked to changes in the spatial and temporal variation of prey from which they sequester VOCs as it has been described for amphibian alkaloids [73, 92, 93]. A plant-to-arthropod, and arthropod-to-frog sequestration sequence is also possible. Moreover, as many of the compounds found in the VOC profile from *S. punctiventris* were found at low intensities (Fig. 1C), a variation higher than the one obtained from alkaloids’ extracts where methanolic extraction is more exhaustive is expected.

From this initial work, new areas of study emerge to perform chemical and behavioral studies with species from the superfamily Dendrobatoidea aimed at 1) contributing to fill the gap of knowledge about the chemical profiles found in cryptic species, 2) broadening the spectrum of compounds analyzed, 3) understanding the function of these chemical signals for intraspecific and interspecific communication, and 4) studying the mechanism of how they are produced. The characterization of the chemical profile from cryptic species needs to be completed and reviewed. To highlight an example of how many gaps need to be studied, not many years ago *E. boulengeri* was used as a negative control for the presence of alkaloids in TLC analysis [48, 67, 94], but GC-MS demonstrated that this species indeed contains alkaloids [65].

To prevent misinterpretations about chemical defences in dendrobatids we suggest avoiding coarse techniques such as TLC. Our results highlight the importance of using GC-MS (or LC-MS) before inferring the absence of alkaloids (or other compounds) in anuran amphibians. Also, we should not forget that besides alkaloids there are many natural products that could have defensive functions. LC-MS was a powerful tool for separating and putatively annotating new metabolites in *P. vitattus* such as bufogenins, bufotenins, and bufadienolides [25]. Furthermore, the combination of HS-SPME with GC-MS offers the possibility of increasing the chemical space sampled, without restricting the extraction and separation methods for alkaloids and diminishing the environmental impact preventing waste residues from organic solvents [95]. Additionally, this method is quicker than conventional alkaloid extraction methodologies [46, 55, 61, 96, 97] and has fewer steps allowing more specimens to be sampled in a finite amount of time. Limitations from this method include difficulties in controlling the thermodynamic equilibrium directly in the field and the differential extraction selectivity for compounds with different polarities and molecular weights [98].

## Conclusions

The chemical analysis from *S. punctiventris* skin employing a head-space solid phase microextraction technique (HS-SPME/GC-MS) provides conclusive evidence about the presence of six amphibian alkaloids and 20 VOCs. This study marks a starting point for 1) conducting the chemical profiling from cryptic species, 2) for incorporating new platforms for the extraction, characterization, and data analysis of a broad spectrum of metabolites, and 3) for exploring the well known toxic/unpalatable, and now we can add possible olfactory function, of the different compounds found in dendrobatids. Previous studies in other cryptic species that suggested apparent inexplicable predator avoidance such as the case of *S. flotator* [47], or malodorous secretions in amphibians such as *A. nocturnus* (or other currently more abundant *Aromobates*) [26], could now be performed with HS-SPME/GC-MS to discover the hidden chemical diversity waiting to be revealed.

## Methods

With the aim of surveying alkaloids and VOCs in *Silverstoneia punctiventris* HS-SPME/GC-MS technique was employed for characterizing their chemical profile*.*

### Collection of animals

**Fig. 3:**
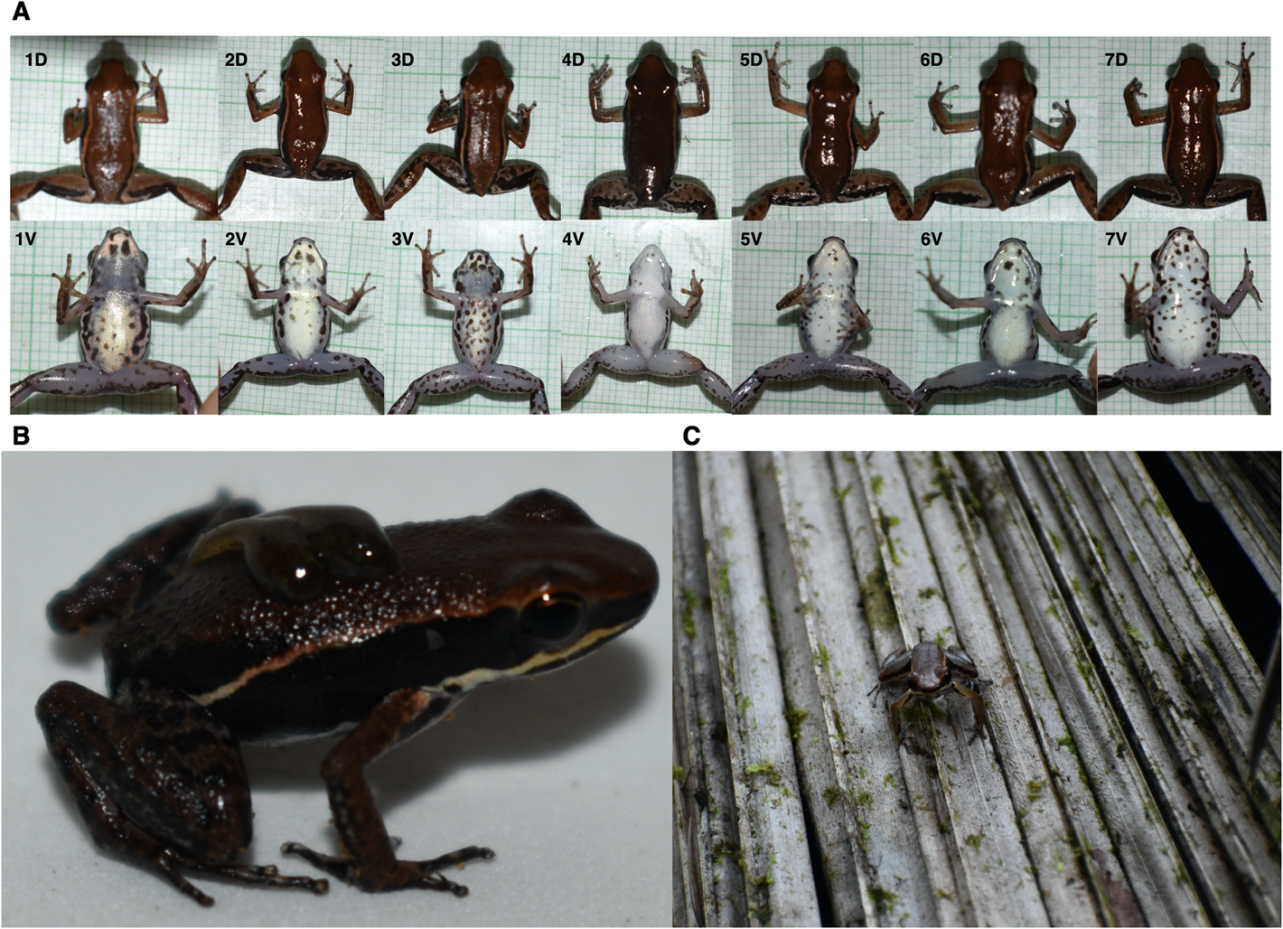
Specimens collected for this study. **A.** Dorsal and ventral view of seven specimens of *S. puctiventris* (D: dorsal view. V: ventral view). Note the variation in the ventral points used as diagnostic traits by Grant & Myers (2013) to define the identity of the species. **B. **Male carrying two tadpoles. **C.** Defence posture during an agonistic encounter. Photos by Pablo Palacios-Rodríguez

*Silverstoneia punctiventris* [99] is a cryptic species endemic to the Chocoan rainforests of Colombia. The species was recognized by the presence of round or elongated black spots scattered throughout the throat, chest, and lateral sides, the diagnostic trait with regard to other species of the genus *Silvestoneia* [99]. Also, the dorsal areas of the hindlimbs had transverse brown bands (Fig. 3).

Eight specimens were captured in the village Puerto Pervel, municipality of Cantón de San Pablo, Department of Chocó, Colombia between March and April of 2019 (Fig. 3A). The collection was authorised by the Colombian Authority for Environmental Licenses (ANLA in Spanish) through the resolution 1177 (Collection of Specimens of Wild Species of Biological Diversity for Non-Commercial Scientific Research Purposes) granted to the Universidad de los Andes. The animals were collected by visual encounter surveys. All animals were collected using a plastic cup to avoid direct manipulation, and later kept in plastic bags with a small amount of water to avoid dehydration. Afterwards, the animals were carefully transported to the Universidad de los Andes for VOC sampling.

### Head-space extraction and gas chromatography

Head-Space Solid Phase Microextraction (HS-SPME) procedure, standardized for sampling the VOCs found on the skin of hylid frogs by Brunetti et al. [30, 31], was adapted for sampling several species of dendrobatids as follows. The frogs were euthanized by immersion in liquid nitrogen. Afterward, two specimens were left at room temperature until thawed. Then, the whole skin of these two specimens was rapidly removed using stainless steel scissors and tweezers, and immediately placed in a ceramic mortar containing liquid nitrogen to minimize the loss of components. Sample homogenization with liquid nitrogen is a widely employed procedure for phytochemical analysis [100] and freezing biological tissues have provided similar recoveries than those obtained by immediate extraction of pyrrolizidine alkaloids in plants or soil [101], but VOC profiles tend to varies between fresh and freeze-thawed samples [102], frozen samples stored by weeks/months [103] or thawed to different temperatures [104]. The skins were homogenized, poured into SPME glass vials of 22 mL (SUPELCO, Palo Alto, CA, USA), and introduced in a thermostatically controlled water bath set to 45 °C. A DVB/CAR/PDMS (SUPELCO, Palo Alto, CA, USA) fiber was inserted into the SPME vial with a sampling time of 40 min. We conducted initial tests to examine the potentiality of HS-SPME for sampling defensive alkaloids such as indolizidines, quinolizidines, or pyrrolizidines in dendrobatids (M. Gonzalez, A. Brunetti, A. Amézquita, M. González-Santoro, P. Palacios-Rodriguez, J. Hernandéz-Restrepo, A. Aksenov, P. Dorrestein, C. Carazzone, unpublished data). The weight of empty vials and vials with wet skin samples were recorded to determine the skin weight to the nearest 0.001 g. Usually, VOC sampling in large-sized dendrobatids can be performed *in vivo* (M. Gonzalez, A. Brunetti, A. Amézquita, M. González-Santoro, P. Palacios-Rodriguez, J. Hernandéz-Restrepo, A. Aksenov, P. Dorrestein, C. Carazzone, unpublished data), but for the minute *S. punctiventris* specimens, we used two complete skins per sampling to guarantee extraction (*S. puctiventris* 1 + 2, *S. puctiventris* 3 + 4, *S. puctiventris* 5 + 6, *S. puctiventris* 7 + 8).

Subsequently, thermal desorption was carried out in the Gas Chromatograph HP 6890 Series equipped with an Agilent Mass Selective Detector 5973 (Agilent Technologies, Palo Alto, CA, USA) at 250 °C in splitless injection mode. The separation was achieved on a BP-5 capillary GC column (30 m × 0.25 mm × 0.25 μm, SGE, Austin, TX, USA) using helium as a carrier gas at a flow rate of 1.0 mL/min. The temperature gradient program started at 40 °C for 3 min, then increased to 100 °C at a rate of 6 °C/min, then again was raised to 200 °C at 4 °C/min, and finally to 300 °C at 20 °C/min; the latter temperature was maintained for 3 min. The GC-MS filament source and the quadrupole temperature were set at 230 and 150 °C, respectively. The electron ionization (EI) source was set at 70 eV, and the mass spectrometer was operated in full scan mode over a mass range from m/z 40 to 300 at a scan rate of 2.0 scan/s. All samples, including linear alkanes, were run under the same chromatographic conditions. Linear alkanes of the series C8–C20 (Sigma, St Louis, MO, USA) were used for the determination of experimental retention indexes (RI exp).

Two specimens were required to make up one biological replicate. For validation of the analysis, four biological replicates of the experimental procedure were performed (Replicate 1: skins of *S. puctiventris* 1 + 2, Replicate 2: skins of *S. puctiventris* 3 + 4, Replicate 3: skins of *S. puctiventris* 5 + 6, Replicate 4: skins of *S. puctiventris* 7 + 8). In addition, to detect trace contaminants from the vial, a blank run was performed before placing the skins. Blank runs of the fiber were used to detect compounds released by the polymers contained in the fiber. These compounds were not taken into account in the analysis of data. All trials, including skin samples, linear alkanes, and blank analyses were run under the same chromatographic conditions.

### Data analysis

To conduct the analysis of the GC-MS data, the profiles with VOCs obtained from the four biological replicates were analyzed with the MSD ChemStation D.02.00.275 (Agilent technologies), and automatic integration using a threshold of 12 units was performed between 0 and 25 min, filtering compounds with peak areas above 50,000 units. Putative annotation of compounds was conducted using NIST MS search 2.0 with the NIST 14 database, through comparison with the fragmentation patterns of alkaloids previously reported by Daly et al. [10], and comparison of experimental retention indexes (RI exp) to theroretical RI (RI theo). In addition to the IUPAC name of the compound, each structure was classified following a standardized chemical taxonomy algorithm and analyzed in a computer program (ClassyFire). This program uses only chemical structures and structural features to automatically assign all known chemical compounds to a taxonomy consisting of > 4800 different categories defined by unambiguous, computable structural rules. Each compound is classified in different levels such as ﻿Kingdom, SuperClass, Class, SubClass, etc. [105]. Amphibian alkaloids, in addition were classified according to Daly et al. alkaloid families [10]. The comparison and details of the annotation process are summarized in the Additional file 2.

Automatic integration results were carefully reviewed, and peak areas were used to construct the matrix in which putative annotated compounds were reported as rows/observations and estimated peak areas as columns/variables. Qualitative comparisons based on peak areas were not affected for the lack of alkaloid internal standards (IS) such as nicotine or decahydroquinoline, previously employed by other researchers that have analyzed methanolic extracts of poison frogs, however it limits semi-quantitative estimations. HS-SPME sampling with IS should be tested and optimized for the unique chemistry found in poison frogs, because matrix effects are very difficult to estimate in living systems subject to headspace sampling with multiphasic equilibria [106]. An alternative tried by some researchers that aimed to characterize the VOC profile of plants was the standard-in-fiber procedure which consists of a short exposition of internal standards on SPME-fibers (e.g. 5 min) before sampling the organism of interest [107].

Defensive/antipredator properties from amphibian alkaloids classes were also included in the matrix using previous information summarized by Santos et al. [22]. Each VOCs was searched on the pherobase database (https://www.pherobase.com/database/compound/compounds-index.php) looking for known behavioral functions. Defence substances, defence pheromones and repellents were selected and included in the matrix. Exported data files of extracted ion chromatograms (EIC) in .csv format were used for the subsequent EIC plotting corresponding to base peaks from putative annotated alkaloids and some VOCs (m/z 152, 166, 135, and 98) (see Fig. 1).

GC-MS runs from this species were converted to. CDF format, uploaded and shared in the MassIVE online repository from GNPS. The specific pipeline recently published for GC-MS data [108] allowed us to run an automatic deconvolution and posterior library search analysis through a community built platform where users share experimental mass spectrometry-based data (derived from LC-MS and now for GC-MS platforms) and libraries for contributing to the democratization of science. We anticipate that GNPS will be a valuable resource to assist researchers working on chemical ecology of dendrobatids and other amphibians in the upcoming years. On the GNPS-GC-MS pipeline, we tracked the retention times from each previous manual annotation and compared the overlap with the automatic annotation list of compounds obtained from GNPS reference libraries of natural products. When available, links of the annotation list for each compound were provided in the Additional files as well as experimental and theoretical retention indexes (Additional file 2).

## Supplementary Information

**Additional file 1.** Title and abstract in Spanish

**Additional file 2 **Detailed annotation process of 33 VOCs from *Silverstoneia punctiventris*. Comparison between manual and automatic annotation specifying IUPAC name of the compound, CAS number for each VOCs, theoretical retention index (RI theo), experimental retention index (RI exp), the difference between theoretical and experimental retention indexes (ΔRI), reference for theoretical RI (Ref RI theo), Classyfire Chemical Superclass, Classyfire chemical Class, Classyfire chemical Subclass/Alkaloid family, reference for behavioral function (Ref BF), retention time in minutes (rtmin), retention time in seconds (rtsec), binary code (Y/N) for selecting if there was a match with GNPS automatic deconvolution process (Match GNPS). When it was a match, in addition, we provided GNPS links of the annotation suspect list for each compound, cosine scores, and balance scores of the selected annotation. Manual annotation was performed using MSD ChemStation D.02.00.275 (Agilent technologies) employing NIST 14 database, and Daly et al. (2005) database. Automatic annotation was performed on the GNPS-GC-MS pipeline employing NIST, Wiley, University of CORSICA databases. When available, links of the annotation list for each compound were provided in the Supplementary material as well as experimental and theoretical retention indexes.

## Data Availability

Title and abstract of this research in Spanish can be accessed in the Additional file 1. The datasets generated and/or analysed during the current study are available in the the MassIVE online repository from the Global Natural Products Social Networking (GNPS), [https://gnps.ucsd.edu/ProteoSAFe/status.jsp?task=2f4aacd2edf0461aa327aa577c553c5d]. The comparison and details of the annotation process and GNPS links are summarized Additional file 2, in the additional files section.
