# Supplementary material for: First characterization of toxic alkaloids and volatile organic compounds (VOCs) in the cryptic dendrobatid Silverstoneia punctiventris
Source: Front Zool. 2021 Aug 26;18:39. doi: 10.1186/s12983-021-00420-1 (PMC8390233; doi:10.1186/s12983-021-00420-1)
Supplement: Supplementary file 1 — Additional file 1. Title and abstract in Spanish [file 12983_2021_420_MOESM1_ESM.docx]

**First characterization of toxic alkaloids and volatile organic compounds (VOCs) in the cryptic dendrobatid *Silverstoneia punctiventris***

**Additional file 1. Title and abstract in Spanish.**

**Primera caracterización de alcaloides tóxicos y compuestos orgánicos volátiles (COVs) en el dendrobátido críptico *Silverstoneia punctiventris***

**Resumen**

**Antecedentes:**

Las ranas venenosas son conocidas por la extraordinaria diversidad de defensas químicas basadas en alcaloides con prometedoras aplicaciones terapéuticas. Sin embargo, el conocimiento actual sobre las defensas químicas en la superfamilia Dendrobatoidea tiene dos fuentes de sesgo. En primer lugar, las especies crípticas de color marrón han sido poco estudiadas en comparación con las especies de coloración llamativa; en segundo lugar, ha habido poco interés en caracterizar metabolitos distintos a alcaloides que median funciones defensivas. En un esfuerzo por contribuir a llenar el vacío de conocimiento sobre las especies crípticas ampliando el espectro de compuestos analizados hemos aplicado la técnica de microextracción en fase sólida de espacio de cabeza acoplada a cromatografía de gases y espectrometría de masas (HS-SPME/GC-MS) para la extracción de alcaloides de anfibios y compuestos orgánicos volátiles (COVs) de *Silverstoneia punctiventris.*

**Resultados:**

Utilizando la piel de 8 especimenes en 4 réplicas biológicas hemos encontrado 33 compuestos diferentes. Veinte de ellos se clasificaron como COVs en 15 clases químicas, incluyendo alcanos, alcoholes, compuestos carbonílicos, metilpiridinas, benzotiazoles, N-alquilpirrolidinas, pirazinas y sesquiterpenoides, algunos de los cuales fueron reportados previamente como repelentes, compuestos de defensa o feromonas de defensa en otros organismos, y como feromonas sexuales en una rana arborícola. Curiosamente, seis de los compuestos restantes fueron identificados como alcaloides previamente reportados en otras ranas dendrobátidas tóxicas/no palatables.

**Conclusiones:**

Este estudio es el primer reporte de alcaloides y COVs encontrados en el género *Silverstoneia*, que ha sido asumido durante décadas como un género no defendido químicamente. Este estudio establece la HS-SPME/GC-MS como una nueva aplicación para un muestreo simultáneo de alcaloides de anfibios y COVs en ranas venenosas, a la vez que abre nuevos preguntas de investigación para evaluar la co-ocurrencia de ambos tipos de compuestos y la posibilidad de investigar la importancia evolutiva de un gradiente de defensa en dendrobátidos, que incluye la defensa olfativa, la no palatabilidad y la toxicidad. Adicionalmente, nuestros resultados muestran que los alcaloides de anfibios podrían tener una función dual (olfativa a distancia, gustativa por contacto) nunca antes explorada ni en *Silverstoneia* ni en ninguna otra especie de dendrobátido.
