# Supplementary material for: First characterization of toxic alkaloids and volatile organic compounds (VOCs) in the cryptic dendrobatid Silverstoneia punctiventris
Source: Front Zool. 2021 Aug 26;18:39. doi: 10.1186/s12983-021-00420-1 (PMC8390233; doi:10.1186/s12983-021-00420-1)
Supplement: Supplementary file 2 — Additional file 2 Detailed annotation process of 33 VOCs from Silverstoneia punctiventris. Comparison between manual and automatic annotation specifying IUPAC name of the compound, CAS number for each VOCs, theoretical retention index (RI theo), experimental retention index (RI exp), the difference between theoretical and experimental retention indexes (ΔRI), reference for theoretical RI (Ref RI theo), Classyfire Chemical Superclass, Classyfire chemical Class, Classyfire chemical Subclass/Alkaloid family, reference for behavioral function (Ref BF), retention time in minutes (rtmin), retention time in seconds (rtsec), binary code (Y/N) for selecting if there was a match with GNPS automatic deconvolution process (Match GNPS). When it was a match, in addition, we provided GNPS links of the annotation suspect list for each compound, cosine scores, and balance scores of the selected annotation. Manual annotation was performed using MSD ChemStation D.02.00.275 (Agilent technologies) employing NIST 14 database, and Daly et al. (2005) database. Automatic annotation was performed on the GNPS-GC-MS pipeline employing NIST, Wiley, University of CORSICA databases. When available, links of the annotation list for each compound were provided in the Supplementary material as well as experimental and theoretical retention indexes. [file 12983_2021_420_MOESM2_ESM.docx]

**First characterization of toxic alkaloids and volatile organic compounds (VOCs) in the cryptic dendrobatid *Silverstoneia punctiventris***

**Additional file 2. Detailed annotation process of 33 VOCs from *Silverstoneia punctiventris*.** Comparison between manual and automatic annotation specifying IUPAC name of the compound, CAS number for each VOCs, theoretical retention index (RI theo), experimental retention index (RI exp), the difference between theoretical and experimental retention indexes (ΔRI), reference for theoretical RI (Ref RI theo), Classyfire Chemical Superclass, Classyfire chemical Class, Classyfire chemical Subclass/Alkaloid family, reference for behavioral function (Ref BF), retention time in minutes (rtmin), retention time in seconds (rtsec), binary code (Y/N) for selecting if there was a match with GNPS automatic deconvolution process (Match GNPS). When it was a match, in addition, we provided GNPS links of the annotation suspect list for each compound, cosine scores, and balance scores of the selected annotation.

| **Compound** | **CAS** | **RItheo** | **Riexp** | **ΔRI** | **Ref RI theo** | **Superclass** | **Class** | **Subclass/Alkaloid family** | **Ref BF** | **rtmin** | **rtsec** | **Match GNPS** | **GNPS link** | **Cosine Score** | **Balance score** |
| --- | --- | --- | --- | --- | --- | --- | --- | --- | --- | --- | --- | --- | --- | --- | --- |
| 3-methylpentane | 96-14-0 | 560 |  |  | (1) | Hydrocarbons | Saturated hydrocarbons | Alkanes |  | 2.27 | 136.22 | N |  |  |  |
| 3-methylbutan-1-ol | 123-51-3 | 735 |  |  | (2) | Organic oxygen compounds | Organooxygen compounds | Alcohols and polyols | (3) | 4.47 | 268.13 | Y | [https://gnps.ucsd.edu/ProteoSAFe/result.jsp?task=2f4aacd2edf0461aa327aa577c553c5d&view=view_all_annotations_DB&show=true#%7B%22main.RT_Query_lowerinput%22%3A%22268%22%2C%22main.RT_Query_upperinput%22%3A%22269%22%7D](https://gnps.ucsd.edu/ProteoSAFe/result.jsp?task=2f4aacd2edf0461aa327aa577c553c5d&view=view_all_annotations_DB&show=true) | 0.97 | 67 |
| hexanal | 66-25-1 | 802 | 800 | 2 | (4) | Organic oxygen compounds | Organooxygen compounds | Carbonyl compounds | (5) | 6.05 | 363.08 | Y | [https://gnps.ucsd.edu/ProteoSAFe/result.jsp?task=2f4aacd2edf0461aa327aa577c553c5d&view=view_all_annotations_DB&show=true#%7B%22main.RT_Query_lowerinput%22%3A%22363%22%2C%22main.RT_Query_upperinput%22%3A%22364%22%7D](https://gnps.ucsd.edu/ProteoSAFe/result.jsp?task=2f4aacd2edf0461aa327aa577c553c5d&view=view_all_annotations_DB&show=true) | 0.89 | 100 |
| 2,4,6-trimethylpyridine | 108-75-8 | 999 | 991 | 8 | http://www.pherobase.com/database/kovats/kovats-detail-collidine.php?isvalid=yes.  (6) | Organoheterocyclic compounds | Pyridines and derivatives | Methylpyridines |  | 11.52 | 691.48 | Y | [https://gnps.ucsd.edu/ProteoSAFe/result.jsp?task=2f4aacd2edf0461aa327aa577c553c5d&view=view_all_annotations_DB&show=true#%7B%22main.RT_Query_lowerinput%22%3A%22690%22%2C%22main.RT_Query_upperinput%22%3A%22693%22%7D](https://gnps.ucsd.edu/ProteoSAFe/result.jsp?task=2f4aacd2edf0461aa327aa577c553c5d&view=view_all_annotations_DB&show=true) | 0.96 | 100 |
| 2-ethylhexan-1-ol | 104-76-7 | 1,034 | 1,028 | 6 | (7) | Lipids and lipid-like molecules | Fatty Acyls | Fatty alcohols |  | 12.57 | 754.10 | Y | [https://gnps.ucsd.edu/ProteoSAFe/result.jsp?task=2f4aacd2edf0461aa327aa577c553c5d&view=view_all_annotations_DB&show=true#%7B%22main.RT_Query_lowerinput%22%3A%22754%22%2C%22main.RT_Query_upperinput%22%3A%22755%22%7D](https://gnps.ucsd.edu/ProteoSAFe/result.jsp?task=2f4aacd2edf0461aa327aa577c553c5d&view=view_all_annotations_DB&show=true) | 0.98 | 85 |
| N,N-dimethyl-1-phenylmethanamine | 103-83-3 |  | 1,040 |  |  | Benzenoids | Benzene and substituted derivatives | Phenylmethylamines |  | 12.89 | 773.51 | Y | [https://gnps.ucsd.edu/ProteoSAFe/result.jsp?task=2f4aacd2edf0461aa327aa577c553c5d&view=view_all_annotations_DB&show=true#%7B%22main.RT_Query_lowerinput%22%3A%22773%22%2C%22main.RT_Query_upperinput%22%3A%22774%22%7D](https://gnps.ucsd.edu/ProteoSAFe/result.jsp?task=2f4aacd2edf0461aa327aa577c553c5d&view=view_all_annotations_DB&show=true) | 0.92 | 100 |
| octan-1-ol | 111-87-5 | 1,070 | 1,069 | 1 | (8) | Lipids and lipid-like molecules | Fatty Acyls | Fatty alcohols | (9) | 13.72 | 823.39 | Y | [https://gnps.ucsd.edu/ProteoSAFe/result.jsp?task=2f4aacd2edf0461aa327aa577c553c5d&view=view_all_annotations_DB&show=true#%7B%22main.RT_Query_lowerinput%22%3A%22823%22%2C%22main.RT_Query_upperinput%22%3A%22824%22%7D](https://gnps.ucsd.edu/ProteoSAFe/result.jsp?task=2f4aacd2edf0461aa327aa577c553c5d&view=view_all_annotations_DB&show=true) | 0.91 | 48 |
| nonanal | 124-19-6 | 1,101 | 1,103 | 2 | (10) | Organic oxygen compounds | Organooxygen compounds | Carbonyl compounds |  | 14.65 | 879.25 | Y | [https://gnps.ucsd.edu/ProteoSAFe/result.jsp?task=2f4aacd2edf0461aa327aa577c553c5d&view=view_all_annotations_DB&show=true#%7B%22main.RT_Query_lowerinput%22%3A%22879%22%2C%22main.RT_Query_upperinput%22%3A%22880%22%7D](https://gnps.ucsd.edu/ProteoSAFe/result.jsp?task=2f4aacd2edf0461aa327aa577c553c5d&view=view_all_annotations_DB&show=true) | 0.98 | 100 |
| 1,2-dimethoxybenzene | 91-16-7 | 1,146 | 1,143 | 3 | http://www.pherobase.com/database/compound/compounds-detail-veratrole.php.  (11) | Benzenoids | Benzene and substituted derivatives | Methoxybenzenes | (12) | 15.85 | 950.80 | Y | [https://gnps.ucsd.edu/ProteoSAFe/result.jsp?task=2f4aacd2edf0461aa327aa577c553c5d&view=view_all_annotations_DB&show=true#%7B%22main.RT_Query_lowerinput%22%3A%22950%22%2C%22main.RT_Query_upperinput%22%3A%22951%22%7D](https://gnps.ucsd.edu/ProteoSAFe/result.jsp?task=2f4aacd2edf0461aa327aa577c553c5d&view=view_all_annotations_DB&show=true) | 0.96 | 100 |
| nonan-1-ol | 143-08-8 | 1,174 | 1,169 | 5 | (7) | Lipids and lipid-like molecules | Fatty Acyls | Fatty alcohols |  | 16.61 | 996.67 | Y | [https://gnps.ucsd.edu/ProteoSAFe/result.jsp?task=2f4aacd2edf0461aa327aa577c553c5d&view=view_all_annotations_DB&show=true#%7B%22main.RT_Query_lowerinput%22%3A%22994%22%2C%22main.RT_Query_upperinput%22%3A%22998%22%7D](https://gnps.ucsd.edu/ProteoSAFe/result.jsp?task=2f4aacd2edf0461aa327aa577c553c5d&view=view_all_annotations_DB&show=true) | 0.93 | 71 |
| 3,5-I **167E**-(3S,5R,8aR)-3-ethyl-5-methyl-1,2,3,5,6,7,8,8a-octahydroindolizine | 94668-38-9 |  | 1,198 |  |  | Organoheterocyclic compounds | Indolizidines | 3,5-I | (13) | 17.43 | 1,046.06 | Y | [https://gnps.ucsd.edu/ProteoSAFe/result.jsp?task=2f4aacd2edf0461aa327aa577c553c5d&view=view_all_annotations_DB&show=true#%7B%22table_sort_history%22%3A%22main.LibMZ_asc%3Bmain.MQScore_dsc%22%2C%22main.%23Scan%23_lowerinput%22%3A%22437%22%2C%22main.%23Scan%23_upperinput%22%3A%22437%22%7D](https://gnps.ucsd.edu/ProteoSAFe/result.jsp?task=2f4aacd2edf0461aa327aa577c553c5d&view=view_all_annotations_DB&show=true) | 0.91 | 100 |
| decanal | 112-31-2 | 1,202 | 1,204 | 2 | (14) | Organic oxygen compounds | Organooxygen compounds | Carbonyl compounds | (15) | 17.63 | 1,057.89 | Y | [https://gnps.ucsd.edu/ProteoSAFe/result.jsp?task=2f4aacd2edf0461aa327aa577c553c5d&view=view_all_annotations_DB&show=true#%7B%22main.RT_Query_lowerinput%22%3A%221057%22%2C%22main.RT_Query_upperinput%22%3A%221058%22%2C%22table_sort_history%22%3A%22main.LibMZ_asc%3Bmain.MQScore_dsc%22%7D](https://gnps.ucsd.edu/ProteoSAFe/result.jsp?task=2f4aacd2edf0461aa327aa577c553c5d&view=view_all_annotations_DB&show=true) | 0.97 | 84 |
| 1,2-benzothiazole | 272-16-2 |  | 1,224 |  |  | Organoheterocyclic compounds | Benzothiazoles | Benzothiazoles |  | 18.25 | 1,094.71 | Y | [https://gnps.ucsd.edu/ProteoSAFe/result.jsp?task=2f4aacd2edf0461aa327aa577c553c5d&view=view_all_annotations_DB&show=true#%7B%22main.RT_Query_lowerinput%22%3A%221094%22%2C%22main.RT_Query_upperinput%22%3A%221095%22%2C%22table_sort_history%22%3A%22main.LibMZ_asc%3Bmain.MQScore_dsc%22%7D](https://gnps.ucsd.edu/ProteoSAFe/result.jsp?task=2f4aacd2edf0461aa327aa577c553c5d&view=view_all_annotations_DB&show=true) | 0.96 | 100 |
| Unknown1 |  |  | 1,113 |  |  |  |  |  |  | 18.55 | 1,112.71 | N |  |  |  |
| 3,5-dimethyl-2-(2-methylbutyl)pyrazine | 56617-70-0 |  | 1,236 |  |  | Organoheterocyclic compounds | Diazines | Pyrazines | (16) | 18.61 | 1,116.49 | Y | [https://gnps.ucsd.edu/ProteoSAFe/result.jsp?task=2f4aacd2edf0461aa327aa577c553c5d&view=view_all_annotations_DB&show=true#%7B%22main.RT_Query_lowerinput%22%3A%221116%22%2C%22main.RT_Query_upperinput%22%3A%221117%22%2C%22table_sort_history%22%3A%22main.LibMZ_asc%3Bmain.MQScore_dsc%22%7D](https://gnps.ucsd.edu/ProteoSAFe/result.jsp?task=2f4aacd2edf0461aa327aa577c553c5d&view=view_all_annotations_DB&show=true) | 0.80 | 100 |
| Unknown2 |  |  | 1,247 |  |  |  |  |  |  | 18.92 | 1,135.37 | N | [https://gnps.ucsd.edu/ProteoSAFe/result.jsp?task=2f4aacd2edf0461aa327aa577c553c5d&view=view_all_annotations_DB&show=true#%7B%22main.RT_Query_lowerinput%22%3A%221135%22%2C%22main.RT_Query_upperinput%22%3A%221136%22%2C%22table_sort_history%22%3A%22main.LibMZ_asc%3Bmain.MQScore_dsc%22%7D](https://gnps.ucsd.edu/ProteoSAFe/result.jsp?task=2f4aacd2edf0461aa327aa577c553c5d&view=view_all_annotations_DB&show=true) | 0.82 | 100 |
| Unknown3 |  |  | 1,255 |  |  |  |  |  |  | 19.18 | 1,150.58 | N | [https://gnps.ucsd.edu/ProteoSAFe/result.jsp?task=2f4aacd2edf0461aa327aa577c553c5d&view=view_all_annotations_DB&show=true#%7B%22main.RT_Query_lowerinput%22%3A%221150%22%2C%22main.RT_Query_upperinput%22%3A%221151%22%7D](https://gnps.ucsd.edu/ProteoSAFe/result.jsp?task=2f4aacd2edf0461aa327aa577c553c5d&view=view_all_annotations_DB&show=true) | 0.83 | 100 |
| Unknown4 |  |  | 1,277 |  |  |  |  |  |  | 19.86 | 1,191.41 | N | [https://gnps.ucsd.edu/ProteoSAFe/result.jsp?task=2f4aacd2edf0461aa327aa577c553c5d&view=view_all_annotations_DB&show=true#%7B%22main.RT_Query_lowerinput%22%3A%221191%22%2C%22main.RT_Query_upperinput%22%3A%221192%22%7D](https://gnps.ucsd.edu/ProteoSAFe/result.jsp?task=2f4aacd2edf0461aa327aa577c553c5d&view=view_all_annotations_DB&show=true) | 0.82 | 100 |
| 1,2,2-triethylpyrrolidine | 89214-92-6 |  | 1,282 |  |  | Organoheterocyclic compounds | Pyrrolidines | N-alkylpyrrolidines | (13) | 20.01 | 1,200.43 | Y | [https://gnps.ucsd.edu/ProteoSAFe/result.jsp?task=2f4aacd2edf0461aa327aa577c553c5d&view=view_all_annotations_DB&show=true#%7B%22main.RT_Query_lowerinput%22%3A%221200%22%2C%22main.RT_Query_upperinput%22%3A%221210%22%7D](https://gnps.ucsd.edu/ProteoSAFe/result.jsp?task=2f4aacd2edf0461aa327aa577c553c5d&view=view_all_annotations_DB&show=true) | 0.84 | 100 |
| Unknown5 |  |  | 1,298 |  |  |  |  |  |  | 20.49 | 1,229.69 | N |  |  |  |
| 1,4-Q **207I**-(1S,4S,9aS)-1-ethyl-4-prop-2-enyl-2,3,4,6,7,8,9,9a-octahydro-1H-quinolizine | 96894-83-6 |  | 1,301 |  |  | Organoheterocyclic compounds | Quinolizines | 1,4-Q | (13) | 20.59 | 1,235.15 | Y | [https://gnps.ucsd.edu/ProteoSAFe/result.jsp?task=2f4aacd2edf0461aa327aa577c553c5d&view=view_all_annotations_DB&show=true#%7B%22main.RT_Query_lowerinput%22%3A%221235%22%2C%22main.RT_Query_upperinput%22%3A%221235.8%22%7D](https://gnps.ucsd.edu/ProteoSAFe/result.jsp?task=2f4aacd2edf0461aa327aa577c553c5d&view=view_all_annotations_DB&show=true) | 0.79 | 100 |
| (3-hydroxy-2,4,4-trimethylpentyl) 2-methylpropanoate |  | 1,387 | 1,374 | 13 | (17) | Organic acids and derivatives | Carboxylic acids and derivatives | Carboxylic acid derivatives |  | 22.79 | 1,367.36 | Y | [https://gnps.ucsd.edu/ProteoSAFe/result.jsp?task=2f4aacd2edf0461aa327aa577c553c5d&view=view_all_annotations_DB&show=true#%7B%22table_sort_history%22%3A%22main.LibMZ_asc%3Bmain.MQScore_dsc%22%2C%22main.RT_Query_lowerinput%22%3A%221367%22%2C%22main.RT_Query_upperinput%22%3A%221368%22%7D](https://gnps.ucsd.edu/ProteoSAFe/result.jsp?task=2f4aacd2edf0461aa327aa577c553c5d&view=view_all_annotations_DB&show=true) | 0.94 | 100 |
| 5,6,8-I **277E**-6,8-dimethyl-5-non-8-enyl-1,2,3,5,6,7,8,8a-octahydroindolizine |  |  | 1,384 |  |  | Organoheterocyclic compounds | Indolizidines | 5,6,8-I | (13) | 23.09 | 1,385.66 | Y | [https://gnps.ucsd.edu/ProteoSAFe/result.jsp?task=2f4aacd2edf0461aa327aa577c553c5d&view=view_all_annotations_DB&show=true#%7B%22main.RT_Query_lowerinput%22%3A%221385%22%2C%22main.RT_Query_upperinput%22%3A%221386%22%2C%22table_sort_history%22%3A%22main.LibMZ_asc%3Bmain.MQScore_dsc%22%7D](https://gnps.ucsd.edu/ProteoSAFe/result.jsp?task=2f4aacd2edf0461aa327aa577c553c5d&view=view_all_annotations_DB&show=true) | 0.83 | 76 |
| 4-(2,6,6-trimethylcyclohexen-1-yl)butan-2-one | 17283-81-7 | 1,440 | 1,440 | 0 | (18) | Lipids and lipid-like molecules | Prenol lipids | Sesquiterpenoids |  | 24.77 | 1,486.21 | Y | [https://gnps.ucsd.edu/ProteoSAFe/result.jsp?task=2f4aacd2edf0461aa327aa577c553c5d&view=view_all_annotations_DB&show=true#%7B%22main.RT_Query_lowerinput%22%3A%221486%22%2C%22main.RT_Query_upperinput%22%3A%221487%22%2C%22table_sort_history%22%3A%22main.LibMZ_asc%3Bmain.MQScore_dsc%22%7D](https://gnps.ucsd.edu/ProteoSAFe/result.jsp?task=2f4aacd2edf0461aa327aa577c553c5d&view=view_all_annotations_DB&show=true) | 0.92 | 100 |
| (E)-4-(2,6,6-trimethylcyclohexen-1-yl)but-3-en-2-one | 79-77-6 | 1,489 | 1,487 | 2 | (19) | Lipids and lipid-like molecules | Prenol lipids | Sesquiterpenoids |  | 26.18 | 1,570.89 | Y | [https://gnps.ucsd.edu/ProteoSAFe/result.jsp?task=2f4aacd2edf0461aa327aa577c553c5d&view=view_all_annotations_DB&show=true#%7B%22main.RT_Query_lowerinput%22%3A%221570%22%2C%22main.RT_Query_upperinput%22%3A%221571%22%2C%22table_sort_history%22%3A%22main.LibMZ_asc%3Bmain.MQScore_dsc%22%7D](https://gnps.ucsd.edu/ProteoSAFe/result.jsp?task=2f4aacd2edf0461aa327aa577c553c5d&view=view_all_annotations_DB&show=true) | 0.83 | 73 |
| 3,5-I **223AB** 3,5-Indolizidine (5E,9Z)-3-butyl-5-propyl-1,2,3,5,6,7,8,8a-octahydroindolizine | 96894-83-6 |  | 1,517 |  | (20) | Organoheterocyclic compounds | Indolizidines | 3,5-I | (13,21) | 27.08 | 1,624.55 | Y | [https://gnps.ucsd.edu/ProteoSAFe/result.jsp?task=2f4aacd2edf0461aa327aa577c553c5d&view=view_all_annotations_DB&show=true#%7B%22main.RT_Query_lowerinput%22%3A%221624%22%2C%22main.RT_Query_upperinput%22%3A%221625%22%2C%22table_sort_history%22%3A%22main.LibMZ_asc%3Bmain.MQScore_dsc%22%7D](https://gnps.ucsd.edu/ProteoSAFe/result.jsp?task=2f4aacd2edf0461aa327aa577c553c5d&view=view_all_annotations_DB&show=true) | 0.92 | 100 |
| 3,5-I **223AB** 3,5-Indolizidine (5E,9E)-3-butyl-5-propyl-1,2,3,5,6,7,8,8a-octahydroindolizine | 150134-99-9 |  | 1,532 |  | (20) | Organoheterocyclic compounds | Indolizidines | 3,5-I | (13,21) | 27.49 | 1,649.35 | Y | [https://gnps.ucsd.edu/ProteoSAFe/result.jsp?task=2f4aacd2edf0461aa327aa577c553c5d&view=view_all_annotations_DB&show=true#%7B%22main.RT_Query_lowerinput%22%3A%221649%22%2C%22main.RT_Query_upperinput%22%3A%221650%22%2C%22table_sort_history%22%3A%22main.LibMZ_asc%3Bmain.MQScore_dsc%22%7D](https://gnps.ucsd.edu/ProteoSAFe/result.jsp?task=2f4aacd2edf0461aa327aa577c553c5d&view=view_all_annotations_DB&show=true) | 0.92 | 100 |
| 3,5-I **223AB** 3,5-Indolizidine (5Z,9Z)-3-butyl-5-propyl-1,2,3,5,6,7,8,8a-octahydroindolizine |  |  | 1,548 |  | (20) | Organoheterocyclic compounds | Indolizidines | 3,5-I | (13,21) | 27.96 | 1,677.37 | Y | [https://gnps.ucsd.edu/ProteoSAFe/result.jsp?task=2f4aacd2edf0461aa327aa577c553c5d&view=view_all_annotations_DB&show=true#%7B%22main.RT_Query_lowerinput%22%3A%221677%22%2C%22main.RT_Query_upperinput%22%3A%221678%22%2C%22table_sort_history%22%3A%22main.LibMZ_asc%3Bmain.MQScore_dsc%22%7D](https://gnps.ucsd.edu/ProteoSAFe/result.jsp?task=2f4aacd2edf0461aa327aa577c553c5d&view=view_all_annotations_DB&show=true) | 0.93 | 100 |
| Unknown6 |  |  | 1,567 |  |  |  |  |  |  | 28.52 | 1,711.09 | N |  |  |  |
| tetradecanal | 124-25-4 | 1,613 | 1,611 | 2 | (19) | Lipids and lipid-like molecules | Fatty Acyls | Fatty aldehydes | (22) | 29.76 | 1,785.77 | Y | [https://gnps.ucsd.edu/ProteoSAFe/result.jsp?task=2f4aacd2edf0461aa327aa577c553c5d&view=view_all_annotations_DB&show=true#%7B%22main.RT_Query_lowerinput%22%3A%221784%22%2C%22main.RT_Query_upperinput%22%3A%221787%22%2C%22table_sort_history%22%3A%22main.LibMZ_asc%3Bmain.MQScore_dsc%22%7D](https://gnps.ucsd.edu/ProteoSAFe/result.jsp?task=2f4aacd2edf0461aa327aa577c553c5d&view=view_all_annotations_DB&show=true) | 0.95 | 78 |
| Unknown7 |  |  | 1,628 |  |  |  |  |  |  | 30.24 | 1,814.61 | N | [https://gnps.ucsd.edu/ProteoSAFe/result.jsp?task=2f4aacd2edf0461aa327aa577c553c5d&view=view_all_annotations_DB&show=true#%7B%22main.RT_Query_lowerinput%22%3A%221814%22%2C%22main.RT_Query_upperinput%22%3A%221815%22%2C%22table_sort_history%22%3A%22main.LibMZ_asc%3Bmain.MQScore_dsc%22%7D](https://gnps.ucsd.edu/ProteoSAFe/result.jsp?task=2f4aacd2edf0461aa327aa577c553c5d&view=view_all_annotations_DB&show=true) | 0.96 | 25 |
| N-butyl-N-(2-oxochromen-3-yl)acetamide |  |  | 1,656 |  |  | Phenylpropanoids and polyketides | Coumarins and derivatives | Coumarins and derivatives |  | 31.02 | 1,861.12 | Y | [https://gnps.ucsd.edu/ProteoSAFe/result.jsp?task=2f4aacd2edf0461aa327aa577c553c5d&view=view_all_annotations_DB&show=true#%7B%22main.RT_Query_lowerinput%22%3A%221861%22%2C%22main.RT_Query_upperinput%22%3A%221862%22%2C%22table_sort_history%22%3A%22main.LibMZ_asc%3Bmain.MQScore_dsc%22%7D](https://gnps.ucsd.edu/ProteoSAFe/result.jsp?task=2f4aacd2edf0461aa327aa577c553c5d&view=view_all_annotations_DB&show=true) | 0.80 | 100 |
| 3-methyl-4-(2,6,6-trimethylcyclohexen-1-yl)but-3-en-2-one | 79-89-0 |  | 1,668 |  |  | Lipids and lipid-like molecules | Prenol lipids | Sesquiterpenoids |  | 31.34 | 1,880.12 | Y | [https://gnps.ucsd.edu/ProteoSAFe/result.jsp?task=2f4aacd2edf0461aa327aa577c553c5d&view=view_all_annotations_DB&show=true#%7B%22main.RT_Query_lowerinput%22%3A%221879%22%2C%22main.RT_Query_upperinput%22%3A%221881%22%2C%22table_sort_history%22%3A%22main.LibMZ_asc%3Bmain.MQScore_dsc%22%7D](https://gnps.ucsd.edu/ProteoSAFe/result.jsp?task=2f4aacd2edf0461aa327aa577c553c5d&view=view_all_annotations_DB&show=true) | 0.81 | 84 |

Manual annotation was performed using MSD ChemStation D.02.00.275 (Agilent technologies) employing NIST 14 database, and Daly et al. (2005) database. Automatic annotation was performed on the GNPS-GC-MS pipeline employing NIST, Wiley, University of CORSICA databases. When available, links of the annotation list for each compound were provided in the Supplementary material as well as experimental and theoretical retention indexes.

**References**

1. Ramírez R, Cava R. Volatile profiles of dry-cured meat products from three different Iberian x Duroc genotypes. J Agric Food Chem. 2007;55(5):1923–31.

2. Fan W, Qian MC. Identification of aroma compounds in Chinese “Yanghe Daqu” liquor by normal phase chromatography fractionation followed by gas chromatography/olfactometry. Flavour Fragr J. 2006;21(2):333–42.

3. Ivarsson P, Henrikson B-I, Stenson JAE. Volatile substances in the pygidial secretion of gyrinid beetles (Coleoptera: Gyrinidae). Chemoecology [Internet]. 1996 Dec;7(4):191–3. Available from: https://linkinghub.elsevier.com/retrieve/pii/0020179086900120

4. Miyazawa M, Tamura N. Components of the essential oil from sprouts ofPolygonum hydropiper L. (‘Benitade’). Flavour Fragr J [Internet]. 2007 May;22(3):188–90. Available from: http://doi.wiley.com/10.1002/ffj.1779

5. Burger BV, Munro Z, Röth M, Geertsema H, Habich A. The chemical nature of the adult defensive secretion of the tip wilter, Elasmopoda valga. Insect Biochem [Internet]. 1986 Jan;16(4):687–90. Available from: https://linkinghub.elsevier.com/retrieve/pii/0020179086900120

6. Radulović NS, Dordević ND, Palić RM. Volatiles of Pleurospermum austriacum (L.) Hoffm. (Apiaceae). J Serbian Chem Soc. 2010;75(12):1653–60.

7. Morteza-Semnani K, Saeedi M, Akbarzadeh M, Moshiri K. The essential oil composition of Onosma microcarpum DC. Flavour Fragr J. 2006;21(2):314–6.

8. Pino JA, Marbot R, Fuentes V. Characterization of volatiles in bullock’s heart (Annona reticulata L.) fruit cultivars from Cuba. J Agric Food Chem. 2003;51(13):3836–9.

9. Kraus B. Effects of honey-bee alarm pheromone compounds on the behaviour of Varroa jacobsoni. Apidologie. 1990;21(2):127–34.

10. Da Silva MHL, Andrade EHA, Zoghbi MDGB, Luz AIR, Da Silva JD, Maia JGS. The essential oils of Lantana camara L. occurring in North Brazil. Flavour Fragr J. 1999;14(4):208–10.

11. Setzer WN, Noletto JA, Lawton RO. Chemical composition of the floral essential oil of Randia matudae from Monteverde, Costa Rica. Flavour Fragr J. 2006;21(2):244–6.

12. Deml R, Dettner K. Biogenic amines and phenolics characterize the defensive secretion of saturniid caterpillars (Lepidoptera: Saturniidae): a comparative study. J Comp Physiol B. 1993;163(2):123–32.

13. Santos JC, Tarvin RD, O’Connell LA. A Review of Chemical Defense in Poison Frogs (Dendrobatidae): Ecology, Pharmacokinetics, and Autoresistance. In: Schulte BA, Goodwin TE, Ferkin MH, editors. Chemical Signals in Vertebrates 13 [Internet]. Cham: Springer International Publishing; 2016. p. 305–37. Available from: http://link.springer.com/10.1007/978-3-319-22026-0

14. Ho C-L, Wang EI-C, Su Y-C. Essential Oil Compositions and Bioactivities of the Various Parts of Cinnamomum camphora Sieb. var. linaloolifera Fujuta. 林業研究季刊 [Internet]. 2009 Jun 1 [cited 2021 Feb 26];31(2):77–95. Available from: https://www.airitilibrary.com/Publication/alDetailedMesh?DocID=16068351-200906-200908060010-200908060010-77-95

15. Burger B V., Marx B, Le Roux M, Oelofsen BW. Characterization of dog repellent factor from cuticular secretion of female yellow dog tick, Haemaphysalis leachi. J Chem Ecol. 2006;32(1):125–36.

16. Guilford T, Nicol C, Rothschild M, Moore B. The biological roles of pyrazines: evidence for a warning odour function. Biol J Linn Soc. 1987;31(2):113–28.

17. Xian Q, Chen H, Zou H, Yin D. Allelopathic activity of volatile substance from submerged macrophytes on Microcystin aeruginosa. Acta Ecol Sin. 2006;26(11):3549–54.

18. Rout PK, Naik SN, Rao YR. Composition of the concrete, absolute, headspace and essential oil of the flowers ofMichelia champaca Linn. Flavour Fragr J [Internet]. 2006 Nov;21(6):906–11. Available from: http://doi.wiley.com/10.1002/ffj.1742

19. Scrivanti LR, Anton AM, Zygadlo JA. Essential oil composition of Bothriochloa Kuntze (Poaceae) from South America and their chemotaxonomy. Biochem Syst Ecol [Internet]. 2009;37(3):206–13. Available from: http://dx.doi.org/10.1016/j.bse.2009.03.009

20. Spande TF, Daly JW, Hart DJ, Tsai YM, Macdonald TL. The structure of gephyrotoxin (GTX) 223AB. Experientia. 1981;37(12):1242–5.

21. Daly JW, Martin Garraffo H, Spande TF. Alkaloids from Amphibian Skins. In 1999. p. 1–161. Available from: https://linkinghub.elsevier.com/retrieve/pii/S0735821099800247

22. Ruano F, Hefetz A, Lenoir A, Francke W, Tinaut A. Dufour’s gland secretion as a repellent used during usurpation by the slave-maker ant Rossomyrmex minuchae. J Insect Physiol. 2005;51(10):1158–64.
